# Healthcare Top Management's Transformational Leadership Behaviors and Nurses' Occupational and Organizational Turnover Intention: On the Role of Work Engagement and Autonomous Motivation

**DOI:** 10.1155/2024/8883038

**Published:** 2024-11-06

**Authors:** Jean-François Gagnon, Claude Fernet, Stéphanie Austin, Sophie Drouin-Rousseau

**Affiliations:** ^1^Département de Gestion des Ressources Humaines, École de Gestion, Université du Québec à Trois-Rivières, Trois-Rivières, Quebec, Canada; ^2^Département de Psychologie, Université de Sherbrooke, Sherbrooke, Quebec, Canada; ^3^École de Psychologie, Université de Moncton, Moncton, New Brunswick, Canada

## Abstract

**Aims:** This study examines the contribution of top management's transformational leadership behaviors on two targets of nurses' turnover intention (organization and occupation) by focusing on the indirect (through vigor and dedication) and conditional indirect associations (involving autonomous motivation as a moderator).

**Background:** Although the issue of nurse turnover has received growing scientific attention, the research is currently silent about the specific targets of turnover intention and more importantly, the potential pathways through which top management's transformational leadership behaviors relate to each target.

**Method:** Cross-sectional data from a sample of 426 French–Canadian nurses and structural equation modeling were used to test the proposed model.

**Results:** Top management's transformational leadership behaviors distinctly predicted organizational and occupational turnover intention through specific nurses' states of engagement. While perceived transformational leadership positively predicted vigor, its indirect associations (via dedication) with organizational and occupational turnover intention depend on nurses' level of autonomous motivation at work.

**Conclusion:** In times of nurse shortage, the present findings provide insights into how and when top management's transformational leadership behaviors relate to nurses' organizational and occupational turnover intention.

**Implications for Nursing Management:** Healthcare organizations are advised to foster top management transformational leadership behaviors and autonomous motivation to sustain the nursing workforce.

## 1. Introduction

Staff turnover in healthcare organizations is a serious concern in several Organization for Economic Co-operation and Development (OECD) countries [1]. In the United States, 23.8% of nurses are reported to leave their jobs after just 1 year of practice [2]. Moreover, a recent American survey indicated that 23% of the registered nurses intended to quit their positions in the next 6 months [3]. In 2013, the World Health Organization (WHO) reported a labor shortage in healthcare facilities of 7.2 million employees [4], a number projected to increase to 18 million by 2030 [5]. This staff shortage, combined with a low retention rate, inevitably exerts pressure on the healthcare system [6]. It goes without saying that this situation generates significant costs, both direct (e.g., recruitment and replacement of staff due to voluntary departures) and indirect (e.g., loss of productivity) for healthcare organizations, thus compromising the safety and quality of the care provided [6, 7].

Studies aiming to understand the issue of turnover widely document turnover intention, a factor which is said to reflect the employee's last cognitive stage before their voluntary departure [8]. More specifically, turnover intention refers to the conscious and deliberate desire to leave an entity [9], such as a healthcare organization or a profession. Among work organization factors of interest, transformational leadership which refers to particular behaviors and practices engaged in by leaders that improve overall performance and outcomes [10] has been shown to play a key role in nurses' turnover intention [11, 12]. These behaviors promote work engagement as they create the conditions required for the appropriation of job resources conducive to positive and adaptative responses, including greater commitment and less turnover intention [13].

Although research in the nursing context has focused more on leadership behaviors of the immediate supervisor, which have been associated with favorable motivational outcomes for nurses (e.g., [12, 14, 15]), top management's behaviors also likely play an important role in nurses' turnover intention. Top management refers to the upper management team within an organization that sets and directs the strategies and policies and makes strategic decisions [16]. In healthcare, this team is responsible for managing the overall operations of the organization and making strategic decisions to ensure the delivery of quality healthcare services [17, 18]. As the decisions of top management pertain, for example, to patient care, resource allocation, infrastructure development, adoption of medical devices, implementation of software and technology, and the formulation of policies and procedures, they play a key role in the day-to-day operations of departments, teams, and units and thus in the work life of nurses. Although we recognize that proximal leaders (e.g., middle or front-line supervisors) are essential in shaping nurses' experience and bringing policies to life, top management appears to play a pivotal leadership role in achieving and maintaining successful changes at every level of the organization [12]. As a first attempt to consider top management leadership, we take the first step to examine the potential motivational pathways by which their behaviors predict nurses' work engagement and turnover intention. We assume that through transformational leadership behaviors (communicating a clear vision, supporting staff development, fostering trust, involvement and cooperation, instilling pride and respect, etc.) top management has the potential to promote nurses' work engagement and prevent their turnover intention.

Aside from the specific, largely understudied, leadership role of healthcare top management, research on the relationship between transformational leadership and nurses' turnover intention remains limited for three main reasons. First, studies neglect the potentially distinct contribution of transformational leadership on various targets of turnover intention, which does not make it possible to assess whether transformational leadership behaviors are as critical to nurses' intention to leave the healthcare organization as they are to their intention to quit the occupation. Second, the role of psychological mechanisms involved in the relationship between top management's transformational leadership behaviors and these two targets of turnover intention (organizational and occupational) remains unknown. Third, the conditions under which these relationships are strengthened or weakened are largely unexplored. Given that work motivation can make employees more or less sensitive to certain sociocontextual factors [19], it could potentially enhance our understanding of how and when transformational leadership behaviors relate to nurses' organizational and occupational turnover intention.

To address this scientific gap, we tested a model which posits that nurses' perception of top management's leadership behaviors is concurrently associated with their organizational and occupational turnover intention through work engagement (i.e., vigor and dedication), especially for nurses with a low level of autonomous motivation at work (i.e., being less inclined to perform their job for pleasure, satisfaction, and interest [20]).

## 2. Theoretical Background

### 2.1. Transformational Leadership

Transformational leadership [10, 21] is characterized by four types of behavior: idealized influence (serving as a role model with whom employees emotionally connect), inspirational motivation (inspiring and motivating employees by sharing values and goals aligned with the mission of the organization), intellectual stimulation (encouraging employees to be creative, innovative, and to question their way of doing things), and individualized consideration (providing a supportive climate and paying attention to nurses' needs, in particular through behaviors and practices aimed at their personal and professional development).

Several studies attest to the contribution of transformational leadership behaviors on various indicators of nurse retention, in particular, organizational turnover intention [12]. Although leadership occurs at all levels of the organization [22], the contribution of healthcare top management's transformational leadership on occupational turnover intention remains to be determined. Simultaneous consideration of the intention to leave the organization and the occupation is therefore important in order to identify the relative contribution of leadership behaviors on turnover intention [23]. Given that top management behaviors are restricted to employees' experience within the organization [21], by facilitating their identification with the organization's goals and values [24], we expect these behaviors to be more strongly associated with nurses' intention to quit the organization rather than the profession.

**Hypothesis 1 .**

Perceived top management's transformational leadership behaviors negatively predict nurses' organizational turnover intention (H1a) and, to a lesser extent, occupational turnover intention (H1b).

### 2.2. Work Engagement

Transformational leadership behaviors are likely to prevent employees' turnover intention, insofar as they create the psychological conditions necessary for their staff to be fully engaged with their jobs [10, 23]. The concept of work engagement seems particularly relevant to account for the motivational role of transformational leadership [25, 26]. More specifically, work engagement [27] describes a positive state of mind specific to the job, characterized by vigor (energy and persistence), dedication (sense of enthusiasm and inspiration), and absorption (full concentration in carrying out tasks). Some research has demonstrated that vigor and dedication are the central dimensions of work engagement [27–29], whereas absorption is rather a consequence [30–32]. Thus, the present study focuses on the energetic (i.e., vigor) and identificatory (i.e., dedication) dimensions of the construct, in accordance with other studies conducted in the nursing context (e.g., [29, 31]). While few studies explore the specific role of top management's transformational leadership in nurses' work engagement, it has been suggested that transformational leadership behaviors among director-level and above positions should exert a stronger influence on nurses' functioning than among manager-level and lower positions [33]. However, the empirical evidence support is still limited to a positive association between nurses' perception of transformational leadership behaviors from the immediate supervisor and their work engagement (e.g., [31, 34, 35]).

**Hypothesis 2 .**

Perceived top management's transformational leadership behaviors positively predict vigor (H2a) and dedication (H2b) in nurses.

According to the job demands–resources (JD–R) model [28], interest in work engagement is partly due to expected motivational outcomes. More specifically, this model implies a motivational process that describes how job resources, including leadership practices, trigger work engagement which, in turn, results in favorable employee attitudes [36, 37]. For instance, Pletzer, Breevaart, and Bakker [26] found that leadership, as a higher order job resource, relates to employees' job performance via work engagement. Since meta-analytic findings indicate that job resources predict work engagement over time [38] and that work engagement relates similarly to motivational outcomes such as job performance, commitment, and turnover intention [39], transformational leadership is expected to spark such a motivational process leading to lower turnover intention. In the nursing context, studies have largely supported this motivational process in explaining organizational turnover intention (e.g., [40, 41]) but without focusing on the top management's leadership behaviors. To our knowledge, no research to date has attempted to determine whether work engagement simultaneously accounts for the association between nurses' perceptions of transformational leadership and organizational and occupational turnover intention. Nevertheless, based on the claims of the motivational process described in the JD–R model and the available findings, we propose that:

**Hypothesis 3 .**

Perceived top management's transformational leadership behaviors are indirectly associated with nurses' organizational turnover intention via vigor (H3a) and dedication (H3b).

**Hypothesis 4 .**

Perceived top management's transformational leadership behaviors are indirectly associated with nurses' occupational turnover intention via vigor (H4a) and dedication (H4b).

### 2.3. Autonomous Motivation at Work

The self-determination theory proposes that the individual's behavioral consequences vary not only according to the strength of motivation but also according to the quality of the motivation [42, 43]. Specifically, the theory distinguishes two main forms of motivation (autonomous and controlled). Autonomous motivation at work refers to the feeling of performing a job by personal choice, mainly for the pleasure and satisfaction it provides or because it is aligned with the individual's personal values [20]. In contrast, controlled motivation refers to efforts made in response to internal (avoiding anxiety or guilt or achieving a sense of self-worth) or external (avoiding constraints or obtaining material or social rewards) pressures. In the present study, we focus on autonomous motivation, as it is more closely related to leadership behaviors than controlled motivation [13, 44].

Several studies have shown that autonomous motivation at work relates to employees' optimal functioning [44, 45]. More specifically, nurses' autonomous motivation has been associated with indicators of psychological health (e.g., work engagement [46]), behavior (e.g., job performance [13]), as well as job attitudes (e.g., commitment and lower organizational and occupational turnover intention [47–49]). While research supports the direct and indirect roles of work motivation [50, 51], some studies indicate that motivation also acts as a moderator between certain work environment factors and indicators of employee functioning (e.g., [19, 52, 53]). For instance, in a study conducted in a school context, Fernet, Gagné, and Austin [19] showed that employees with low autonomous motivation were more sensitive and responsive to the quality of interpersonal relationships with their colleagues, which predicted burnout over time. Consistent with the principle of behavioral plasticity [54], these findings suggest that employees with a high level of autonomous motivation, who autonomously value and internalize their tasks as part of their professional identity [20], are less affected by external factors. This is likely because they are more naturally inclined to perform elements of the job that are aligned with their interests, values, and professional goals. In contrast, for employees with lower levels of autonomous motivation at work, certain aspects of the work environment, including leadership behaviors, should have a boosting motivational effect on their work engagement. This should lead them to be more contingent on the behaviors of their supervisor to be fully engaged with their job. Although the leadership of top management appears to play a central role in nurses' professional life [33, 55], no study to date has examined the motivational conditions under which its behaviors are more likely to relate to nurses' psychological health (i.e., vigor and dedication) and, in turn, their job attitudes (i.e., organizational and occupational turnover intention). Based on the available theory and empirical findings, we propose the following hypothesis.

**Hypothesis 5 .**

Nurses' autonomous motivation at work moderates the relationship between perceived transformational leadership behaviors and turnover intention (organization and occupational) through work engagement (H5a: vigor and H5b: dedication). More specifically, the associations between transformational leadership behaviors and work engagement dimensions are expected to be greater when nurses' level of autonomous motivation is low.

## 3. Methods

### 3.1. Design and Sample

This cross-sectional study is part of a broader research program on the health and well-being of nurses in the context of professional integration in Québec, Canada. The recruitment involved a total of 3800 nurses contacted by email through their professional association (Quebec Professional Nursing Association). They were informed about the research objectives, told that participation was voluntary and confidential, and sent a link to the online questionnaire to be completed. Among the 657 nurses who filled the online questionnaire (completion rate of 77.9%; response rate of 16.7%), 426 met the inclusion (i.e., holding a position that involves daily direct contact with patients) and exclusion (i.e., holding a management or counseling position and inability to read and understand French) criteria. Our convenience sample size is within the range of participants typically used, and recommended as a minimum, in applied CFA-SEM research (e.g., [56]), and consistent with the sample size used in recent moderated mediation analysis among nurses (e.g., *n* = 290 [57]; *n* = 419 [58]). Moreover, our analyses revealed no indications that the sample size might have been insufficient (i.e., proper convergence, statistically significant effects, and reasonable standard errors). Most participants were female (87.6%) and held permanent (76.4%) and full-time (50.5%) positions. The average age was 29.3 years (SD = 7.8). On average, the nurses had 3.24 years (SD = 2.95) of experience in the nursing profession and 2.25 years (SD = 1.41) of experience at the same healthcare organization. In terms of work schedule, 30.3% of the participants worked an evening schedule, 17% worked during the day, 24.5% worked at night, and 28% had a varied schedule.

### 3.2. Measures

All measures were administered in French. Although only the autonomous motivation scale has been formally validated in French (see [45]), the validity and fidelity of the French–Canadian version of these measures have been supported in prior studies (e.g., [13, 47, 48, 59]). Psychometric properties for the present study are presented in Table 1.

#### 3.2.1. Transformational Leadership

The Global Transformational Leadership (GTL [60]) Scale was used to assess nurses' perception of top management's leadership behaviors. The measure includes seven items (e.g., encourages us and recognizes our work) rated on a five-point scale ranging from 1 (*never*) to 5 (*frequently or always*). In the present study, the internal consistency coefficient is satisfactory (α = 0.94) and similar to those reported in other studies on the leadership behaviors of the supervisors [13, 61].

#### 3.2.2. Work Engagement

Six items from the Utrecht Work Engagement Scale (UWES-9 [62]) were administered, using a seven-point scale ranging from 0 (*never*) to 6 (*every day*), to assess the two main dimensions of work engagement, namely, vigor (e.g., at my work, I feel bursting with energy; three items; α = 0.88) and dedication (e.g., I am enthusiastic about my job; three items; α = 0.90). The internal consistency coefficients observed are similar to those reported by other researchers [59, 63].

#### 3.2.3. Autonomous Motivation

The Multidimensional Work Motivation Scale (MWMS [64]) was used to measure nurses' autonomous motivation at work. Respondents scored on a seven-point scale from 1 (*not at all for this reason*) to 7 (*exactly for this reason*), the primary reasons for performing their job. Following the procedure commonly used in the SDT-based research [46, 47], and consistent with the operationalization of construct [45], we used identified regulation (three items; e.g., because this job has a personal significance for me) and intrinsic motivation (three items; e.g., because my work is stimulating) to assess autonomous motivation. The internal consistency coefficient is satisfactory (α = 0.84) and consistent with values obtained in prior studies (e.g., [47, 59]).

#### 3.2.4. Organizational Turnover Intention

Intention to leave the healthcare organization was measured using four items (e.g., I am thinking about leaving my current healthcare facility) adapted from [65]. Respondents provided ratings on a seven-point scale ranging from 1 (*strongly disagree*) to 7 (*strongly agree*). The internal consistency coefficient is satisfactory (α = 0.87) and in line with the values reported in previous studies (e.g., [49]).

#### 3.2.5. Occupational Turnover Intention

Three items adapted from [65] were used to assess occupational turnover intention (e.g., “I am thinking about leaving the nursing profession”). Each item was scored on a seven-point scale from 1 (*strongly disagree*) to 7 (*strongly agree*). The internal consistency coefficient is satisfactory (α = 0.92) and similar to previously reported values (e.g., [49]).

### 3.3. Statistical Analysis

Models were tested through structural equation modeling using a robust maximum likelihood estimator (MLR) in Mplus 8.10 [66]. Missing data ranged from 2.52% to 20.41% with higher rates for organizational (18.58%–20.41%) and occupational (18.58%–19.27%) turnover intention which may be attributable to participants' sensitivity about these particular variables. They were handled with the full information maximum likelihood (FIML) algorithm which is appropriate given the estimated amount of missing data [67]. To determine the goodness-of-fit of these models, various fit indices that are sample size independent [68–70] were used as follows: The comparative fit index (CFI), the Tucker–Lewis index (TLI), and the root mean square error of approximation (RMSEA). Values between 0.90 and 0.95 for the CFI and TLI and values smaller than 0.08 and 0.06 for the RMSEA indicate acceptable and excellent levels of fit [70].

First, confirmatory factor analysis (CFA) were performed to assess a measurement model. Second, we tested a structural equation model (SEM) in which autonomous motivation and transformational leadership predict components of work engagement (vigor and dedication) and turnover intention (organizational and occupational). This predictive model was tested to confirm the adequate representation of the data, given that fit indices are not available for the latent variable interaction tests. Third, tests of latent variable interactions were conducted using the SEM approach to estimate latent moderation effects (LMS), with standardized values obtained directly in Mplus [71]. Although the method proposed by Hayes and Preacher [72] is commonly used to detect significant interactions, it relies on an arbitrary choice of values (e.g. ± 1 SD) for the moderator to probe the interaction effect. To circumvent this limitation, we plotted the effect of each value of the moderator on the coefficient values of the relationship between our independent variable (transformational leadership) and dependent variable (dedication) using the Johnson–Neyman technique [73–75]. This technique provides a region of significance that is determined by the lower and upper bound of the confidence interval (the effect is not significant for any value of the moderator when 0 lies within the confidence interval). Finally, bootstrapped confidence interval estimates of the indirect associations were calculated (with 5000 bootstrap resamples) to confirm the significance of the mediation.

### 3.4. Ethical Considerations

Ethical approval was obtained from the research ethics board of the corresponding author's institution (CER-11-174-07-02.13).

## 4. Results

### 4.1. Preliminary Analyses

CFA results showed satisfactory fit indices: *Χ*^2^ = 491.702 (284), CFI = 0.960, TLI = 0.955, and RMSEA = 0.041 (0.035; 0.048). The estimated parameters (saturation coefficients and uniqueness) are presented in Table 1 and revealed well-defined and differentiated constructs. As presented in Table 2, latent correlations were significant and in the expected direction.

### 4.2. Main Analyses

**Figure 1:**
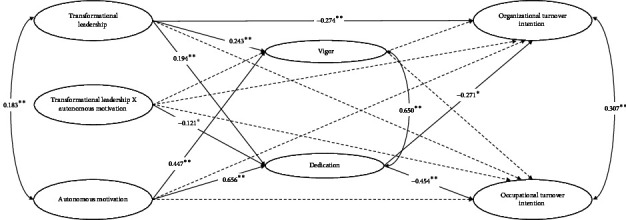
Results of the structural equation model. *Note:* standardized coefficients are reported. ⁣^∗∗^*p* < 0.01; ⁣^∗^*p* < 0.05; dotted lines represent nonsignificant effects.

The fit indices of the predictive model remained excellent: *Χ*^2^ = 491.702 (284), CFI = 0.960, TLI = 0.955, and RMSEA = 0.041 (0.035; 0.048). As presented in Table 3 and Figure 1, the results showed that perceived transformational leadership behaviors are negatively associated with organizational turnover intention (β = −0.274; *p* < 0.01) but not significantly with occupational turnover intention (β = −0.085; *p*=0.198). These results support Hypothesis 1a but lead us to reject Hypothesis 1b.

In addition, the results showed that perceived transformational leadership behaviors are positively associated with work vigor (β = 0.243; *p* < 0.01) and dedication (β = 0.194; *p* < 0.01), thus supporting Hypotheses 2a and 2b. Although the results did not reveal a significant relationship between vigor and organizational turnover intention (β = −0.043; *p*=0.659), they showed a significant negative relationship between dedication and organizational turnover intention (β = −0.271; *p* < 0.05), thus refuting Hypothesis 3a but confirming Hypothesis 3b. Also, the results showed a negative, but not significant, relationship between vigor and occupational turnover intention (β = 0.050; *p*=0.519), as well as a significant negative relationship between dedication and occupational turnover intention (β = −0.454; *p* < 0.01), thus refuting Hypothesis 4a but confirming Hypothesis 4b.

**Figure 2:**
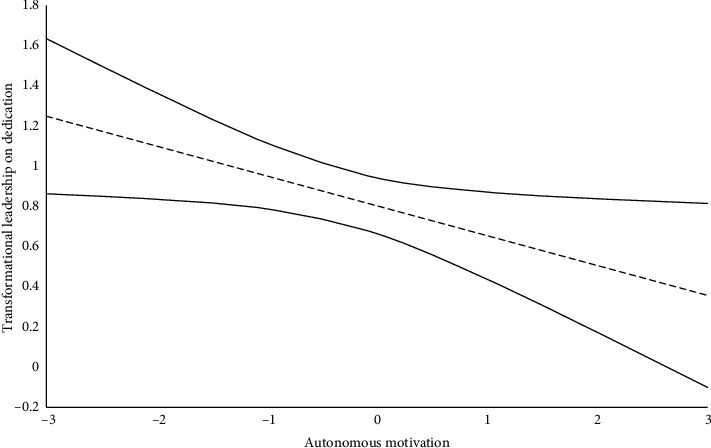
Johnson–Neyman analysis of the association between transformational leadership and dedication at different levels of autonomous motivation.

Our results did not reveal a significant interaction effect between autonomous motivation and perceived transformational leadership behaviors in predicting vigor (β = −0.030; *p*=0.648), organizational turnover intention (β = 0.026; *p*=0.641), and occupational turnover intention (β = 0.030; *p*=0.704), leading us to reject Hypothesis 5a. However, in line with Hypothesis 5b, the interaction between autonomous motivation and transformational leadership behaviors in predicting dedication was significant (β = −0.121; *p* < 0.05).  Figure 2 depicts, at different levels of autonomous motivation, the association between transformational leadership and dedication. The results indicate that the association between perceived transformational leadership behaviors and work dedication is stronger for nurses characterized by lower levels of autonomous motivation. Bootstrapped analyses confirmed the conditional indirect association between transformational leadership behaviors and organizational (low, *b* = −0.154 [−0.375; −0.030]; mean, *b* = −0.095 [−0.218; −0.018]; high, *b* = −0.036 [−0.118; 0.002]) and occupational (low, *b* = −0.177 [−0.364; −0.062]; mean, *b* = −0.109 [−0.216; −0.042]; high, *b* = −0.041 [−0.110; 0.002]) turnover intention, via dedication, across low and mean levels of autonomous motivation.

## 5. Discussion

The aim of this study was to examine the link between top management's transformational leadership behaviors and nurses' occupational and organizational turnover intention by focusing on the indirect (through vigor and dedication) and conditional indirect associations (involving autonomous motivation as a moderator). Our results show that transformational leadership behaviors distinctly predict organizational and occupational turnover intention through specific states of engagement. While perceived transformational leadership is positively associated with vigor, its indirect association (via dedication) with organizational and occupational turnover intention depends on nurses' level of autonomous motivation at work. These findings contribute to theory and extend research on transformational leadership, work engagement, motivation, and turnover intention in three main ways.

### 5.1. Top Management's Leadership Behaviors and Specific Targets of Turnover Intention

In line with previous studies, our results indicate that transformational leadership behaviors are associated with employee well-being [76] and favorable job attitudes [77]. However, our results extend prior research, not only by highlighting the role of healthcare top management's transformational leadership behaviors but also by delineating different turnover intention pathways. While perceived top management's transformational leadership behaviors relate to nurse's vigor and dedication at work, only dedication appears to be indirectly associated with turnover intention. Interestingly, our results suggest a full indirect association for occupational turnover intention, but a partial indirect association for organizational turnover intention. This finding lends support to the idea that top management may not only be related to their employees' state of mind [76] but that they also play a more direct role in their organizational turnover intention. This suggests that through leadership behaviors and practices with which they intend to encourage their employees to go beyond their personal interests for the good of the organization, top management could influence, in addition to their contribution to employee well-being, their staff's turnover intention to the healthcare organization. From a leadership perspective, this may highlight the importance of top management's leadership behaviors in helping develop a culture of commitment to the organization. Thus, our results contribute to theories of transformational leadership by underlying the important role of top management leaders and by determining potential pathways by which their behaviors affect distinct targets of turnover intention.

### 5.2. The Distinct Role of Vigor and Dedication

While demonstrating the need to consider organizational and occupational turnover intention separately, our results suggest that only work dedication is involved in the proposed relationships. Our findings show that vigor and dedication are independent and play distinct roles in predicting turnover intention. Interestingly, top management's leadership behaviors appear to be especially important to foster nurses' dedication (possibly because such behaviors give the work a sense of purpose) but not sufficient to sustain high levels of vigor at work (possibly due to the highly demanding nature of the profession). These results are consistent with Bakker et al.'s [78] theoretical proposition that vigor and dedication constitute different components of work engagement. Vigor is theorized to be at the center of an energetic process and dedication of an identification process. The transformational leadership behaviors of top management could thus contribute more to the identification process in which nurses identify strongly, with pride and enthusiasm, with their work, which would reduce organizational and occupational turnover intention. These results therefore add to the JD–R model [27, 36] by identifying some of the psychological mechanisms liable to predict turnover intention. Nonetheless, future studies would do well to consider the role of other agents in these processes, as behaviors of proximal leaders (e.g., front-line supervisors) may be at play in the energetic process. As we can only speculate on these psychological processes (associations do not imply causation), it would be informative for future studies to delve further into causal inference. Nevertheless, our study provides the conceptual and empirical bases on which to build a more solid understanding of how top management's leadership behaviors relate to organizational and occupational turnover intention.

### 5.3. The Moderating Role of Autonomous Motivation

The identification process observed in our results depends on employees' level of autonomous motivation at work. The contribution of leadership behaviors on dedication was found to be higher for nursing staff with lower levels of autonomous motivation. These results are interesting insofar as top management is a key figure in the workplace who can influence the employee identification process, especially for those for whom work motivation is characterized by a lesser sense of self-determination. As evidenced by other studies [13], these employees' responsiveness to leadership behaviors could therefore nurture a strong sense of work identification, which would reduce organizational and occupational turnover intention. In line with the principle of behavioral plasticity [54], our results extend its scope to employees' motivational resources in their adaptation to top management's behavior. Contrary to our prediction, we did not find support for the conditional indirect association of transformational leadership behaviors on turnover intention, via vigor, across each level of autonomous motivation. One potential explanation for this finding may lie in the energetic nature of this state of engagement. Although some studies have shown that employees with low levels of autonomous motivation are more affected by external factors [19, 52], health-impairment responses (such as a state of exhaustion) may be more likely to occur and have a greater impact on turnover intention.

### 5.4. Limitations

Despite the contributions detailed above, our study has certain limitations that should be noted. First, our study relies on self-report measures that may be subject to social desirability and self-report bias. Future research that uses triangulation by integrating multisource (e.g., peer leadership assessment) and objective (e.g., actual departure) data would enhance the validity of our findings. Still, since data were collected from a single source, it raises the possibility of common variance bias. To address this concern, we tested a CFA model that included an additional method factor (i.e., single-method–scale-score approach; [79]) to estimate the proportion of shared variance in the model. Results showed that the method factor accounts for 8.3% of the total variance, which suggests that the model is not biased by the method factor [79, 80]. The observed moderating role of autonomous motivation limits this possibility, given that the presence of common variance would probably not strengthen the relationship between top management's leadership behaviors and dedication solely for participants who reported lower levels of motivation. Second, the study is based on a cross-sectional design, which does not allow causal relations between variables to be established. Although some studies support some of the proposed indirect associations, via work engagement [34, 81], for example between leadership behaviors and turnover intention [61], the possibility of reciprocal or inverse relationships between certain variables should not be excluded [82]. Thus, future studies should further validate our findings using longitudinal designs to achieve a better understanding of the psychological mechanisms involved. Third, although we relied on a proven theoretical framework (JD–R model; [27, 36]) to determine the choice of psychological mechanisms to include, our analysis relies on a limited number of variables. While the synergistic contribution of top management's leadership behaviors and autonomous motivation at work is better documented, further research should broaden this understanding to other mechanisms, such as trust in leaders or other agents in the work environment [83]. In addition, the simultaneous consideration of leadership of multiple agents (top managers, middle managers, and front-line supervisors) will provide insights into their relative contribution. Finally, our study is exclusively based on a sample of nurses from the province of Quebec, Canada. Thus, our results need to be replicated with nurses in other countries and cultures, as well as with employees of other industries.

### 5.5. Practical Implications

Our study suggests courses of action aimed at reducing nurses' turnover intention both at the organizational and occupational levels. Through its different effects on each target of turnover intention, our findings suggest that courses of intervention should consider top management's leadership behaviors. Training in transformational leadership [84] proves to be a promising avenue that healthcare organizations and both initial and continuing training programs may wish to promote. Along with the efforts, it may be useful to target antecedents of transformational leadership which generally fall into three categories: leader qualities (e.g., self-efficacy, values, and emotional intelligence), organizational characteristics (e.g., fairness), and characteristics of the leader's staff (e.g., cohesion and cooperation) [85]. Any action aimed at strengthening one of these dimensions should contribute to improving top management's transformational leadership behaviors. Finally, since nurses' autonomous motivation at work interacts with transformational leadership behaviors, it may be relevant to enhance the quality of employee motivation. In addition to being largely beneficial to job commitment [49, 50] and optimal functioning at work [44, 45], autonomous motivation should limit, to a certain extent, the contingency of leadership behaviors to the expression of favorable states of work engagement. To do this, research has clearly evidenced that workplaces that tend to cultivate the satisfaction of employees' psychological needs (autonomy, competence, and relatedness) contribute not only to their autonomous motivation but also to their optimal functioning [51]. Such initiatives could contribute to fostering work engagement and to reducing nurses' organizational and occupational turnover intention.

## 6. Conclusion

This study underscores the importance of healthcare top management's transformational leadership in predicting nurses' turnover intention and provides valuable insights into the potential pathways at play. Our findings indicate that top management's leadership behaviors are distinctly associated with nurses' occupational and organizational turnover intention through specific states of engagement. While perceived leadership behaviors positively predicted vigor, their indirect association (via dedication) with organizational and occupational turnover intention depends on nurses' level of autonomous motivation at work. Organizational efforts to cultivate transformational leadership behaviors and promote autonomous motivation could constitute promising steps to sustain the nursing workforce.

## Figures and Tables

**Table 1:** Saturation of standardized factors (*λ*) and their uniqueness (*δ*).

Items	TL *λ*	AM *λ*	VI *λ*	DE *λ*	OTI *λ*	OcTI *λ*	*δ*
Transformational leadership							
Item 1	0.739						0.453
Item 2	0.878						0.230
Item 3	0.896						0.198
Item 4	0.923						0.149
Item 5	0.864						0.253
Item 6	0.736						0.458
Item 7	0.846						0.285
ω	0.945						
Autonomous motivation							
Item 1		0.823					0.322
Item 2		0.858					0.264
Item 3		0.823					0.322
Item 4		0.735					0.460
Item 5		0.347					0.879
Item 6		0.464					0.785
ω		0.844					
Vigor							
Item 1			0.736				0.458
Item 2			0.928				0.139
Item 3			0.902				0.187
ω			0.894				
Dedication							
Item 1				0.883			0.220
Item 2				0.959			0.080
Item 3				0.749			0.438
ω				0.901			
Organizational turnover intention							
Item 1					0.879		0.228
Item 2					0.572		0.673
Item 3					0.872		0.239
Item 4					0.837		0.299
ω					0.874		
Occupational turnover intention							
Item 1						0.875	0.234
Item 2						0.833	0.307
Item 3						0.948	0.101
ω						0.917	

*Note:* ω, omega coefficient; *λ*, saturation of standardized factors; *δ*, uniqueness.

Abbreviations: AM, autonomous motivation; DE, dedication; OcTI, occupational turnover intention; OTI, organizational turnover intention; TL, transformational leadership; VI, vigor.

**Table 2:** Latent standardized correlations between variables.

Latent variables	2	3	4	5	6
1. Transformational leadership	0.184	0.317	0.289	−0.355	−0.210
2. Autonomous motivation	—	0.495	0.700	−0.222	−0.402
3. Vigor		—	0.760	−0.318	−0.370
4. Dedication			—	−0.361	−0.511
5. Organizational turnover intention				—	0.434
6. Occupational turnover intention					—

*Note:* Correlations are all significant (*p* < 0.01).

**Table 3:** Results of the structural equation model.

Predictors	Vigor		Dedication		Organizational turnover intention		Occupational turnover intention	
	*b* (SD)	β	*b* (SD)	β	*b* (SD)	β	*b* (SD)	β
Without interaction								
TL	0.233 (0.060)⁣^∗∗^	0.234⁣^∗∗^	0.205 (0.056)⁣^∗∗^	0.166⁣^∗∗^	−0.483 (0.108)⁣^∗∗^	−0.268⁣^∗∗^	−0.094 (0.076)	−0.076
AM	0.452 (0.071)⁣^∗∗^	0.452⁣^∗∗^	0.824 (0.074)⁣^∗∗^	0.670⁣^∗∗^	0.085 (0.148)	0.047	−0.106 (0.111)	−0.085
VI					−0.071 (0.177)	−0.039	0.068 (0.093)	0.055
DE					−0.419 (0.166)⁣^∗^	−0.286⁣^∗^	−0.475 (0.111)⁣^∗∗^	−0.471⁣^∗∗^
*R*^2^	0.298 (0.049)⁣^∗∗^		0.517 (0.055)⁣^∗∗^		0.200 (0.042)⁣^∗∗^		0.271 (0.055)⁣^∗∗^	
Interactions								
TL	0.243 (0.065)⁣^∗∗^	0.243⁣^∗∗^	0.237 (0.064)⁣^∗∗^	0.194⁣^∗∗^	−0.496 (0.111)⁣^∗∗^	−0.274⁣^∗∗^	−0.105 (0.084)	−0.085
AM	0.447 (0.072)⁣^∗∗^	0.447⁣^∗∗^	0.803 (0.071)⁣^∗∗^	0.656⁣^∗∗^	0.077 (0.148)	0.042	−0.114 (0.113)	−0.092
TL × AM	−0.030 (0.066)	−0.030	−0.149 (0.068)⁣^∗^	−0.121⁣^∗^	0.047 (0.100)	0.026	0.037 (0.096)	0.030
VI					−0.078 (0.178)	−0.043	0.062 (0.098)	0.050
DE					−0.400 (0.171)⁣^∗^	−0.271⁣^∗^	−0.459 (0.121)⁣^∗∗^	−0.454⁣^∗∗^
*R*^2^	0.299 (0.049)⁣^∗∗^		0.530 (0.051)⁣^∗∗^		0.205 (0.044)⁣^∗∗^		0.271 (0.056)⁣^∗∗^	

*Note:* β, standardized regression coefficient; *b*, nonstandardized regression coefficient; *R*^2^, variance.

Abbreviations: AM, autonomous motivation; DE, dedication; SD, standard deviation; TL, transformational leadership; VI, vigor.

⁣^∗^*p* < 0.05.

⁣^∗∗^*p* < 0.01.

## Data Availability

Data are available on request from the authors.
